# Disrupted tongue microbiota and detection of nonindigenous bacteria on the day of allogeneic hematopoietic stem cell transplantation

**DOI:** 10.1371/journal.ppat.1008348

**Published:** 2020-03-09

**Authors:** Saori Oku, Toru Takeshita, Toshiko Futatsuki, Shinya Kageyama, Mikari Asakawa, Yasuo Mori, Toshihiro Miyamoto, Jun Hata, Toshiharu Ninomiya, Haruhiko Kashiwazaki, Yoshihisa Yamashita

**Affiliations:** 1 Section of Preventive and Public Health Dentistry, Division of Oral Health, Growth and Development, Faculty of Dental Science, Kyushu University, Fukuoka, Japan; 2 Section of Geriatric Dentistry and Perioperative Medicine in Dentistry, Faculty of Dental Science, Kyushu University, Fukuoka, Japan; 3 OBT Research Center, Faculty of Dental Science, Kyushu University, Fukuoka, Japan; 4 Department of Medicine and Biosystemic Science, Kyushu University Graduate School of Medical Sciences, Fukuoka, Japan; 5 Department of Epidemiology and Public Health, Graduate School of Medical Sciences, Kyushu University, Fukuoka, Japan; 6 Center for Cohort Studies, Graduate School of Medical Sciences, Kyushu University, Fukuoka, Japan; University of Toronto, CANADA

## Abstract

Disruption of the intestinal microbiota caused by intensive chemotherapy, irradiation and antibiotics can result in development of severe gut graft-versus-host disease and infectious complications, leading to poorer outcomes among allogeneic hematopoietic stem cell transplantation (allo-HSCT) recipients. Although the oral cavity is also densely colonized by indigenous microorganisms, the bacterial composition in allo-HSCT recipients remains unclear. We determined the tongue microbiota composition of 45 patients with hematological disorders on the day of transplantation and compared them to 164 community-dwelling adults. The V1–V2 regions of the 16S rRNA gene sequences demonstrated that the allo-HSCT recipients had less diverse and distinct microbiota from that of community-dwelling adults. The full-length 16S rRNA gene sequences identified 146 bacterial taxa in the microbiota of allo-HSCT recipients, of which 34 bacterial taxa did not correspond to bacteria primarily inhabiting the oral cavity deposited in the expanded Human Oral Microbiome Database. Notably, the detection of *Staphylococcus haemolyticus* and/or *Ralstonia pickettii* was significantly associated with a higher risk of mortality during the follow-up period. These results demonstrate that the oral cavity of allo-HSCT recipients is colonized by a disrupted microbiota on the day of transplantation and suggest that detection of specific nonindigenous taxa could be a predictor of transplant outcome.

## Author summary

Allogeneic hematopoietic stem cell transplantation (allo-HSCT) recipients are subjected to intensive chemotherapy, irradiation and antibiotics which could affect the intestinal as well as oral microbiota. We employed full-length 16S rRNA gene sequencing analysis with high taxonomic resolution using a third-generation sequencer, PacBio Sequel, and determined the bacterial composition of the tongue microbiota of allo-HSCT recipients after conditioning regimens. This comprehensive molecular approach identified 34 taxa uncommon in the oral cavity, which constituted 0–99.4% (median, 0.27%) of each tongue microbiota. Of them, *Staphylococcus haemolyticus* and *Ralstonia pickettii* were frequently found in allo-HSCT recipients, and their detection was significantly associated with a higher risk of mortality during the follow-up period. These results suggest that careful attention should be given to the bacterial composition of the disrupted oral microbiota in allo-HSCT recipients.

## Introduction

Allogeneic hematopoietic stem cell transplantation (allo-HSCT) is a curative treatment option for various hematological malignancies and inherited hematopoietic disorders [1, 2]. In order to eradicate residual malignant cells, as well as immunocompetent cells to ensure engraftment of infused donor cells, allo-HSCT recipients undergo a conditioning regimen including intensive chemotherapy and/or total body irradiation [3], resulting in mucosal injury. They require broad-spectrum antibiotics until neutrophil recovery in order to prevent and treat bacterial penetration into the bloodstream through the damaged mucosal barrier. Long-term use of broad-spectrum antibiotics can seriously affect the indigenous microbiota, which in the steady-state contributes to maintaining homeostasis among microorganisms or between the microorganisms and the host [4]. Dysbiosis of the gastrointestinal tract in allo-HSCT recipients was reportedly associated with the increased risk of bacteremia [5]; severe graft-versus-host disease (GVHD)[6, 7]; disease relapse [8]; and, ultimately, inferior overall survival [9].

The oral cavity is also densely colonized by diverse microorganisms constituting the indigenous microbiota, which serves as a defensive barrier against the establishment of pathogenic bacteria [10]; thus, disruption of the oral microbiota could lead to colonization by exogenous pathogenic bacteria in the oral cavity. The bacterial populations shed from intraoral surfaces are constantly transported to the gastrointestinal and respiratory tracts via saliva and could contribute to systemic health. The preparative regimen prior to allo-HSCT and broad-spectrum antibiotics could also alter the oral microbiota. In fact, a recent study reported that the denaturing gradient gel electrophoresis profiles of oral mucosal microbiota drastically changed after allo-HSCT in six recipients, especially in recipients who received glycopeptide antibiotics in combination with ß-lactam [11]. Another recent study using 16S rRNA gene sequencing analysis indicated a decrease in identified bacterial taxa and the presences of unique bacterial taxa in oral microbiota in allo-HSCT based on longitudinal observation at three timepoints of four aplastic anemia patients who received allo-HSCT [12]. However, the oral bacterial composition of allo-HSCT recipients and its association with transplant outcome have not been fully elucidated.

In this study, we collected the tongue microbiota, a dominant source of the bacterial population in saliva [13–15], from 45 allo-HSCT recipients suffering from hematological disorders on the day of transplantation. The composition of the microbiota was compared with that of community-dwelling adults by using 16S rRNA gene sequencing analysis with a next-generation sequencer, and further determined based on full-length 16S rRNA gene sequences obtained from a third-generation long-read sequencer with a high taxonomic resolution. We also sought to elucidate the bacterial taxa that produce an impact on the transplant outcome.

## Results

This study investigated the bacterial composition of the tongue microbiota collected from 45 adult allo-HSCT recipients [19 females and 26 males aged 36–69 years; mean ± standard deviation (SD): 52.7 ± 10.3 years] on the day of transplantation. Underlying hematological diseases were as follows: 23 patients with acute myelogenous leukemia, 10 with malignant lymphoma, 5 with acute lymphoblastic leukemia, 5 with myelodysplastic syndrome, and 2 with other conditions (Table 1). Of 45 patients, 20 patients underwent myeloablative conditioning regimens that contained 12 Gy total body irradiation or 12.8–mg/kg busulfan, whereas the remaining 25 patients underwent fludarabine-based reduced-intensity conditioning regimens that contained low-dose irradiation (2–4 Gy). Prophylactic oral levofloxacin was initiated at least 6 days before transplantation and was switched to other broad-spectrum antibiotics (e.g., cefepime, tazobactam/piperacillin, carbapenems) if the patients developed febrile neutropenia.

**Table 1:** Baseline characteristics of the study population.

	No. of patients
Parameter	(n = 45)
Median age, yr (range)	53 (36–69)
Sex	
Female/Male	19/26
Underlying disease	
Acute myelogenous leukemia	23
Malignant lymphoma	10
Acute lymphoblastic leukemia	5
Myelodysplastic syndrome	5
Primary myelofibrosis	1
Aplastic anemia	1
Risk status at transplantation	
Standard/High	22/23
Conditioning regimen	
Myeloablative/Reduced intensity	20/25
Total body irradiation	
None	10
Low-dose/High-dose	21/14
Graft source	
Bone marrow	21
Peripheral blood	20
Cord blood	4
HLA parity	
Matched/Mismatched	24/21
Antibiotic use during conditioning	
Prophylactic/Treatment use	19/26
GvHD prophylaxis	
CIs+sMTX	32
CIs+MMF±PTCy	9
Others	4
Prior transplantation	
Yes/No	6/39
Median number of teeth (range)	27 (5–32)
Bacteremia until day+100	
Yes/No	12/33
Oral mucositis	
Yes/No	28/17
Grade 2 to 4	25
Grade 3 to 4	15
Acute GvHD	
Yes/No	25/20
Grade II to IV	15
Grade III to IV	4

Abbreviation: GvHD, graft-versus host disease, CIs, calcineurin inhibitors; MTX, methotrexate; MMF, mycophenolate mofetil; PTCy, post-transplant cyclophosphamide

**Fig 1:**
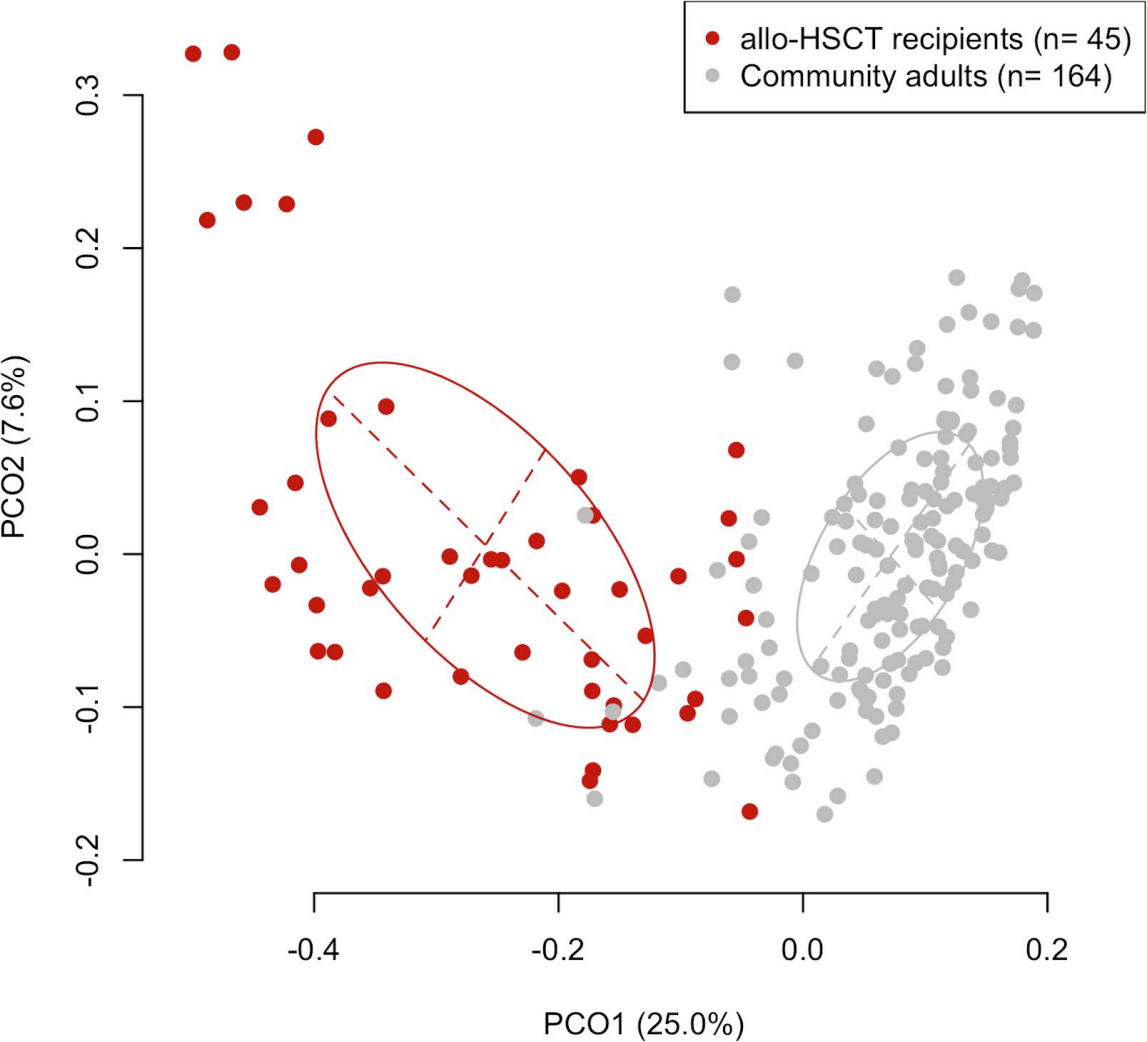
Unweighted UniFrac plot of microbiota composition between community-dwelling adults and allo-HSCT patients. The ellipses cover 67% of the samples belonging to each sample type.

**Fig 2:**
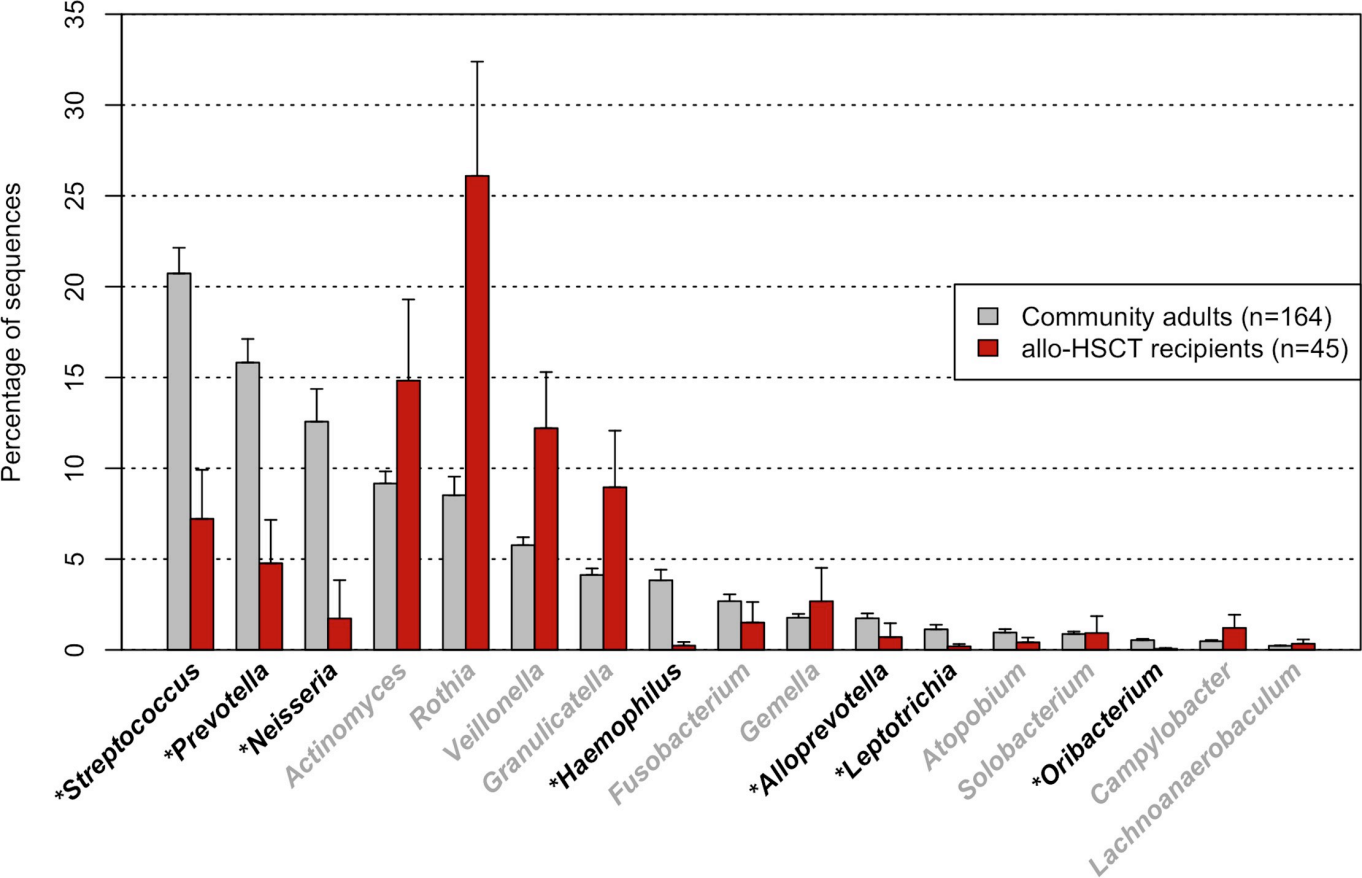
Relative abundances of predominant bacterial genera in the tongue microbiota of community-dwelling adults and allo-HSCT patients. Only 17 bacterial genera commonly (>98%) present in community-dwelling adults are shown. *P <0.05 in a Wilcoxon rank-sum test followed by *P* value adjustment for multiple comparison.

First, the overall composition of the oral microbiota of allo-HSCT recipients was compared with that of 164 age-matched (mean ± SD: 55.0 ± 10.8 years) community-dwelling adults including 93 females and 71 males based on the results of partial 16S rRNA gene sequencing (V1–V2 region) following rarefaction to 2,000 reads per sample to correct the unequal number of sequences per sample. A principal coordinate analysis (PCoA) plot based on the unweighted UniFrac metric demonstrated that the bacterial composition of the allo-HSCT recipients was distinct from that of the community-dwelling adults (Fig 1). Although the predominant bacterial genera in the community-dwelling adults were also present in the allo-HSCT recipients, those genera, such as *Streptococcus*, *Prevotella*, and *Neisseria*, were present at a significantly lower relative abundance in the allo-HSCT recipients (Fig 2). All three alpha diversity indices indicated that the microbiota of the allo-HSCT recipients was much less diverse than that of the community-dwelling adults (Table 2). In contrast, a substantial number of species-level operational taxonomic units (OTUs), absent in community-dwelling adults, were found in the microbiota of the allo-HSCT recipients (S1 Table). These OTUs were barely detectable even in the large number (3,077,343) of all obtained reads of the community-dwelling adults (S1 Table) which almost covers the bacterial taxa in their microbiota (S1 Fig). Most of these OTUs did not correspond to bacterial taxa for which the primary body site is denoted as “Oral” in the expanded Human Oral Microbiome Database (eHOMD) [16].

**Table 2:** Alpha diversity indices of the tongue microbiota of 164 community-dwelling adults and 45 allogeneic hematopoietic stem cell transplantation (allo-HSCT) recipients on transplantation date in V1-V2 regions of 16S rRNA gene sequencing analysis.

	Community-dwelling adults (n = 164)	allo-HSCT recipients (n = 45)	*P*-value^a^
Number of OTU	90.9 ± 18.3	41.1 ± 15.6	<0.001
Phylogenetic diversity	6.9 ± 1.1	4.0 ± 1.1	<0.001
Shannon diversity index	3.2 ± 0.3	2.1 ± 0.6	<0.001

Values with errors are mean ±SD.

^a^Wilcoxon rank-sum test.

To identify the nonindigenous taxa detected in the oral cavity of the allo-HSCT recipients, we further employed full-length 16S rRNA gene sequencing analysis with a high taxonomic resolution by using a third-generation sequencer, PacBio Sequel (Pacific Biosciences, Menlo Park, CA, USA). A number of accurate full-length 16S rRNA gene sequences (about 1,500 bases) were obtained by the use of the circular consensus sequence (CCS) technique, which repeatedly determines the template DNA sequences based on long sequencing potential, although the lower throughput (as compared with the short-read sequencers) only allowed us to determine the microbiota of allo-HSCT recipients. A total of 152,040 high-quality CCS reads corresponding to 1,014 distinct full-length 16S rRNA gene sequences were obtained for the 45 recipients (mean ± SD, 3378 ± 1533 reads; range, 770–7198 reads), after a quality filtering and denoising procedure using R and DADA2. The rarefaction curve for the number of unique sequences almost approached a plateau in each sample (S2 Fig). The relative abundances of each bacteria and alpha diversity in full-length 16S rRNA gene sequencing analysis differed but highly correlated with those in V1–V2 region of 16S rRNA gene sequencing analysis (S3 Fig).

Of the obtained 16S rRNA gene sequences, 934 distinct sequences containing 139,923 reads (92.0%) were assigned to the 112 oral indigenous taxa (“Oral” was included in their “Body Site” status in eHOMD) deposited in the eHOMD [16], with ≥ 98.5% identity. Most of the microbiota on the tongue of allo-HSCT recipients were composed of taxa common in the oral cavity, including *Rothia mucilaginosa*, *Granulicatella adiascens* and *Veillonella dispar* (S4 Fig). On the other hand, as many as 12,101 reads corresponding to 78 sequences, were unassigned and corresponded to 17 non-oral taxa in eHOMD and 17 bacterial taxa deposited in the SILVA database. The remaining 2 sequences containing 16 reads could not be identified using this database, so we determined their taxonomies up to the genus level. Of the 34 identified non-oral taxa, 15 were found in the microbiota of multiple allo-HSCT recipients (Table 3). These non-oral bacteria obtained predominance in the disrupted oral microbiota of some allo-HSCT recipients (Table 3); in particular, *Staphylococcus haemolyticus* comprised more than 40% of the microbiota in three patients.

**Table 3:** Nonindigenous bacteria identified from the tongue microbiota found in allo-HSCT recipients on the transplantation date in full-length 16S rRNA gene analysis.

	No. of subjects with each taxon	Relative abundance (%)	
Bacterial taxa		Mean	Max
*Ralstonia pickettii* (854)	13	0.06±0.18	1.06
*Staphylococcus haemolyticus* (120)	8	4.96±19.29	95.41
uncultured organism (HQ813300)	8	0.14±0.57	3.75
*Streptococcus thermophilus* (152)	6	1.09±6.33	42.49
*Lactobacillus casei* (568)	3	0.14±0.91	6.14
*Enterococcus durans* (880)	2	0.49±2.84	18.91
uncultured *Prevotella* sp. (AM420032)	2	0.06±0.38	2.53
*Enterococcus faecalis* (604)	2	0.05±0.34	2.28
*Streptococcus pneumoniae* (734)	2	0.03±0.14	0.70
*Stenotrophomonas maltophilia* (663)	2	0.01±0.09	0.57
*Acinetobacter ursingii* (APQB01000014, APQC01000015, APQC01000022)	2	0.01±0.07	0.50
uncultured organism (HQ771651)	2	0.01±0.07	0.44
*Xanthomonas arboricola* (AJTL01000224)	2	0.01±0.06	0.39
uncultured bacterium (GQ129984)	2	0.01±0.03	0.15
*Ottowia* sp. (894)	2	0±0.02	0.11
uncultured bacterium (EU937980)	1	0.17±1.13	7.61
*Corynebacterium tuberculostearicum* (077)	1	0.06±0.41	2.74
uncultured *Pseudomonas* sp. (HM152712)	1	0.04±0.27	1.84
*Pseudomonas monteilii* (BBIS01000088)	1	0.02±0.16	1.08
*Enterobacter hormaechei* (634)	1	0.02±0.14	0.96
uncultured bacterium (HE681344)	1	0.02±0.10	0.69
*Finegoldia magna* (662)	1	0.01±0.08	0.52
*Atopobium vaginae* (814)	1	0.01±0.04	0.30
uncultured *Bacteroidetes* bacterium (KC169767)	1	0.01±0.04	0.25
*Ochrobactrum anthropi* (544)	1	0±0.03	0.21
*Delftia acidovorans* (023)	1	0±0.02	0.17
Genus *Alloprevotella* dOTU842	1	0±0.02	0.17
Genus *Leptotrichia* dOTU925	1	0±0.03	0.17
*Leuconostoc lactis* (AEOR01001150)	1	0±0.02	0.14
uncultured bacterium (AM277332)	1	0±0.02	0.13
*Neisseria macacae* (099)	1	0±0.02	0.11
*Clostridium butyricum* (X68178)	1	0±0.01	0.10
*Selenomonas* sp. oral taxon 136 (CP014239)	1	0±0.01	0.09
*Pseudomonas stutzeri* (477)	1	0±0.01	0.04

Values with errors are mean ±SD.

^a^Taxon ID in expanded Human Oral Microbiome database (triple digits) or GenBank number is given in parentheses following bacterial name.

**Fig 3:**
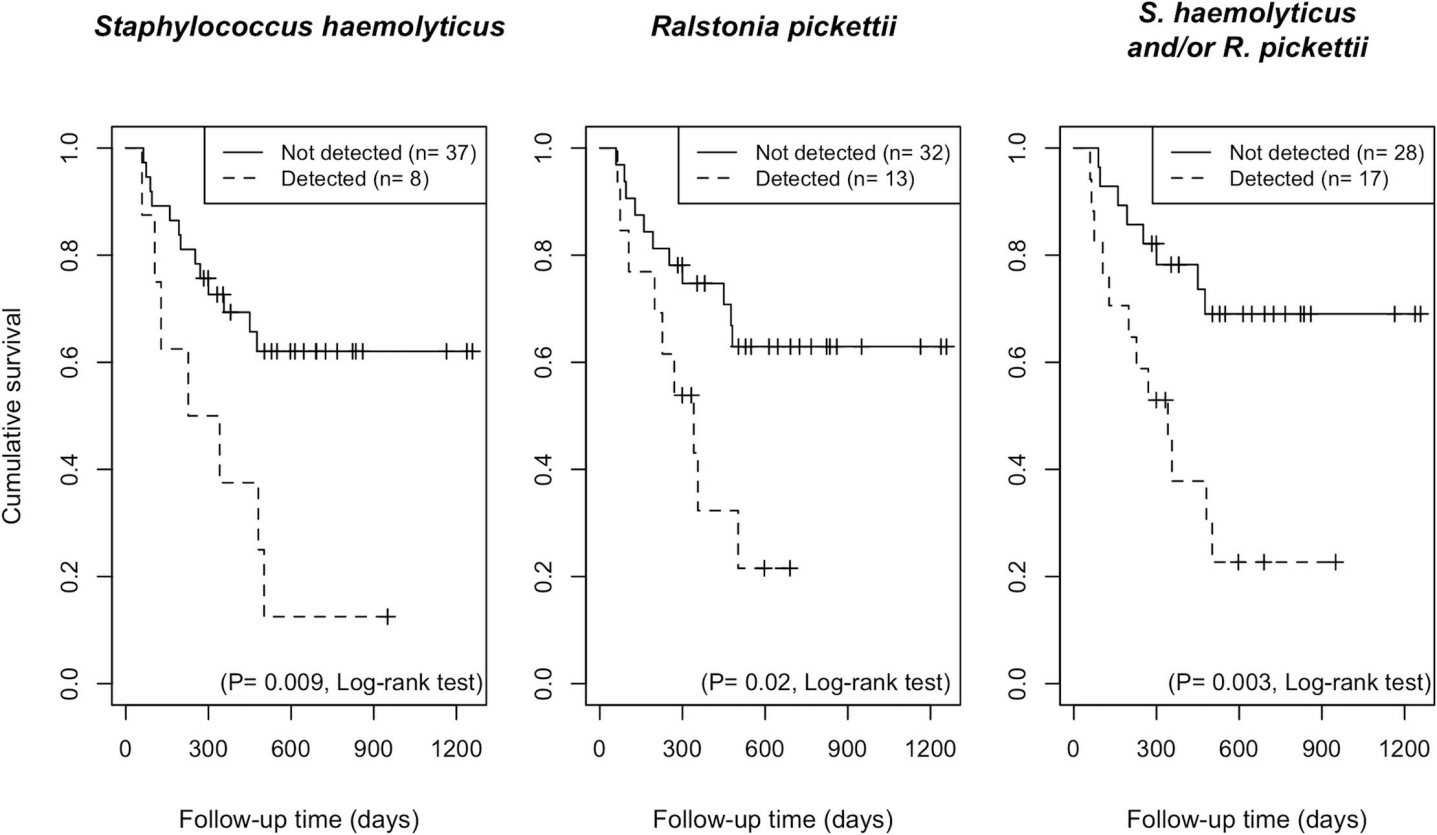
Survival plots of the patients with detection or no detection of *Staphylococcus haemolyticus* and *Ralstonia pickettii* on the tongue generated by the Kaplan–Meier method. Significant differences were determined using a log-rank test.

The detection of the top 4 non-oral bacteria (observed in more than 5 cases) present in the oral cavity on the day of stem cell infusion, was not associated with clinical parameters, including incidence of oral mucositis, bacteremia through 100 days after transplantation, or acute GvHD (S2 Table). In contrast, *Staphylococcus haemolyticus* or *Ralstonia pickettii*, as well as either or both taxa were associated with a significantly higher risk of all-cause mortality at 692 days of the median follow-up period (*P* = 0.009, *P* = 0.02 and *P* = 0.003, respectively, log-rank test; Fig 3). The estimated one-year overall survival rate among recipients positive for *Staphylococcus haemolyticus* and/or *Ralstonia pickettii* was 37.8% (95% confidence interval (CI), 19.7%–72.3%), whereas that of patients without detection of either of these taxa, was 78.2% (95% CI, 64.2%–95.3%). Of 17 patients positive for *Staphylococcus haemolyticus* and/or *Ralstonia pickettii*, 12 had died of progressive disease (n = 8), GvHD (n = 3), or infection (n = 1), whereas 8 of 28 patients without detection of these taxa, had died of progressive disease (n = 5), GvHD (n = 1), or infection (n = 2).

Among other potential covariates, disease risk stratification prior to transplantation was significantly associated with the survival rate during the follow-up period in a univariate Cox proportional hazards regression analysis (Hazard Ratio (HR) = 5.1, 95% CI = 1.8–14.2; *P* = 0.001), along with detection of either/both of the two taxa (HR = 3.5, 95% CI = 1.4–8.6; *P* = 0.006) (Table 4). A multivariate model incorporating these two factors demonstrated that detection of either/both of the two taxa remained statistically significant independent of the risk status (adjusted HR = 2.5, 95% CI = 1.0–6.4; *P* = 0.04: Table 4). We constructed another model incorporating additional use of antibiotics with the detection of either/both of the two taxa considering their potential relationship. Detection of either/both of the two taxa was also significantly associated with a higher risk of mortality in this model, while no significant relationship was observed between additional use of antibiotics and higher risk of mortality after the adjustment (Table 4).

**Table 4:** Univariate and multivariate associations of the detection of *Staphylococcus haemolyticus* and/or *Ralstonia pickettii* with the high risk of mortality at the follow-up period in Cox proportional hazard regression models.

	Univariate		Multivariate			
			Model 1		Model 2	
	HR (95% CI)	*P*-value	aHR (95% CI)	*P*-value	aHR (95% CI)	*P*-value
Detection of either/both of the two taxa	3.5 (1.4–8.6)	.006	2.5 (1.0–6.4)	.04	3.1 (1.0–9.2)	.03
Age	1.0 (0.9–1.0)	.26				
Underlying disease (leukemia vs other)	1.2 (0.4–3.2)	.66				
Risk status at transplantation	5.1 (1.8–14.2)	.001	4.1 (1.4–11.7)	.008		
Conditioning regimens						
Myeloablative	(reference)					
Reduced intensity	1.3 (0.5–3.2)	.54				
Total body irradiation						
None	(reference)					
Low dose	1.2 (0.2–2.1)	.62				
High dose	1.8 (0.1–1.7)	.30				
Antibiotics use during conditioning						
Prophylactic use	(reference)					
Additional use for treatment	2.3 (0.8–6.5)	.09			1.2 (0.3–4.2)	.70
Graft source						
Peripheral blood	(reference)					
Bone marrow	1.3 (0.5–3.4)	.53				
Cord blood	1.1 (0.2–5.4)	.86				
HLA parity						
Matched	(reference)					
Mismatched	1.3 (0.5–3.1)	.53				
GvHD prophylaxis						
CIs+sMTX	(reference)					
CIs+MMF±PTCy	1.4 (0.5–4.0)	.47				
Others	1.5 (0.3–7.0)	.54				

Abbreviation: HR, hazard ratio; aHR, adjusted hazard ratio; GvHD, graft-versus host disease; CIs, calcineurin inhibitors; MTX, methotrexate; MMF, mycophenolate mofetil; PTCy, post-transplant cyclophosphamide.

We further analyzed the characteristics of patients with and without detection of these bacteria and sought causative variables associated with this difference in transplant outcome between the two groups (Table 5). Additional use of antibiotics for febrile neutropenia and/or infections to prophylactic oral levofloxacin was significantly relevant for the detection of these bacteria. Furthermore, a higher detection rate was observed in patients receiving multidrug regimens, rather than those receiving single cephem for the treatment of febrile neutropenia and/or infections (S3 Table). The PCoA plot based on unweighted UniFrac distance also showed that the additional antibiotics use, rather than conditioning regimens and underlying diseases, had an impact on the overall bacterial composition (S5 Fig).

**Table 5:** Relationship between the detection of *Staphylococcus haemolyticus* and/or *Ralstonia pickettii* and the baseline characteristics of the recipients.

	Either/both of the two taxa		
	Detected	Not detected	
	(n = 17)	(n = 28)	*P*-value^a^
Age (Mean ±SD)	54±10	51±10	0.46^a^
No. (%) of female	9 (52.9)	10 (35.7)	0.35^b^
Underlying disease			0.73^b^
Acute myelogenous leukemia	8 (47.0)	15 (53.5)	
Malignant lymphoma	5 (29.4)	5 (17.8)	
Acute lymphoblastic leukemia	1 (5.8)	4 (14.2)	
Myelodysplastic syndrome	2 (11.7)	3 (10.7)	
Primary myelofibrosis	0 (0)	1 (3.5)	
Aplastic anemia	1 (5.8)	0 (0)	
Prior transplantation	2 (11.7)	4 (14.2)	1.00^b^
Risk status at transplantation			0.06^b^
Standard	5 (29.4)	17 (60.7)	
High	12 (70.5)	11 (39.2)	
Conditioning regimens			0.37^b^
Myeloablative	6 (35.2)	14 (50.0)	
Reduced intensity	11 (64.7)	14 (50.0)	
Antibiotic use during conditioning			0.001^b^
Prophylactic use	2 (11.7)	17 (60.7)	
Additional use for treatment	15 (88.2)	11 (39.2)	
Total body irradiation			0.72^b^
None	4 (23.5)	6 (21.4)	
Low dose	9 (52.9)	12 (42.8)	
High dose	4 (23.5)	10 (35.7)	
Graft source			0.07^b^
Peripheral blood	11 (64.7)	10 (35.7)	
Bone marrow	4 (23.5)	16 (57.1)	
Cord blood	2 (11.7)	2 (7.1)	
HLA parity			0.23^b^
Matched	7 (41.1)	17 (60.7)	
Mismatched	10 (58.8)	11 (39.2)	
GvHD prophylaxis			0.41^b^
CIs+sMTX	10 (58.8)	22 (78.5)	
CIs+MMF±PTCy	5 (29.4)	4 (14.2)	
Others	2 (11.7)	2 (7.1)	

^a^Student’s t-test.

^b^Fisher’s exact test.

Abbreviation: GvHD, graft-versus host disease; CIs, calcineurin inhibitors; MTX, methotrexate; MMF, mycophenolate mofetil; PTCy, post-transplant cyclophosphamide.

## Discussion

This study demonstrated that the indigenous oral microbiota of allo-HSCT recipients was disrupted at the time point of stem cell transplantation after receiving intensive chemotherapy, radiation, and antibiotics. The lower diversity of the oral microbiota, as well as the change in components, among allo-HSCT recipients as compared with that in community-dwelling adults was confirmed (Table 2 and Fig 1). The bacterial genera predominantly observed in the oral microbiota of community-dwelling adults such as *Streptococcus*, *Neisseria* and *Prevotella*, occupied a significantly lower proportion of the microbiota in allo-HSCT recipients (Fig 2). Instead, taxa uncommon in the oral cavity were detected or even dominated in the microbiota of several allo-HSCT recipients (Table 3 and S1 Table).

Use of the long-read sequencer with the CCS technique, which can provide numerous accurate full-length 16S rRNA gene sequences [17, 18] identified as many as 34 bacterial taxa not corresponding to bacteria primarily inhabiting the oral cavity deposited in eHOMD [16] in the tongue microbiota of allo-HSCT recipients (Table 3). Nonetheless, there remains the possibility that they were merely transient bacteria present in the oral cavity at the time of sample collection. However, although this cross-sectional study could evaluate only a snapshot of the tongue microbiota of allo-HSCT recipients on the day of transplantation, 15 of 34 non-oral bacteria were detected in the tongue swab samples of multiple allo-HSCT recipients (Table 3). In addition, 12 taxa were identified as predominant in the tongue microbiota with maximum relative abundances >1% (Table 3), suggesting their detection was not just contamination. Many of these taxa, such as *Ralstonia pickettii* [19], *Staphylococcus haemolyticus* [20], *Enterococcus faecalis* [21], *Stenotrophomonas maltophilia* [22], and *Acinetobacter ursingii* [23] reportedly had natural or acquired resistance to antibiotics. Thus, detection of these pathological bacteria was quite reasonable in the microbiota of allo-HSCT recipients treated with long-term broad-spectrum antibiotics and/or antifungal agents during the peritransplant period. Whether their detection results from their overgrowth or drastic decrease of other indigenous bacteria or both remains unknown in this study, because the 16S rRNA gene sequencing analysis only allows qualitative consideration on the bacterial community, or relative abundances of bacteria. Nevertheless, in any of these cases, it is a remarkable phenomenon indicating disruption of indigenous microbiota which serves as a defensive barrier against infection.

Of the uncommon taxa found in allo-HSCT patients, the detection of *Staphylococcus haemolyticus* and *Ralstonia pickettii* on the day of transplantation could be a powerful predictor of transplant outcome (Fig 3). *Staphylococcus haemolyticus*, a coagulase-negative staphylococci (CNS), is a member of the normal skin microbiota [20]. With its ability to acquire multidrug resistance, this microorganism has reportedly been the second most frequently isolated CNS, following *Staphylococcus epidermidis*, especially as sourced from the bloodstream [24]. *Ralstnoia pickettii*, a non-fermenting Gram-negative bacillus, is not a major pathogen but has been isolated from a wide variety of clinical specimens, including blood and sputum, or even from contaminated fluids used for patient care [19]. Aspiration or ingestion of these bacteria might be associated with poorer outcome of allo-HSCT recipients. However, the major cause of mortality was progressive diseases, and no patient died of the complications associated with detected *Staphylococcus haemolyticus* or *Ralstonia pickettii* in the present study. The incidence of bacteremia until 100 days after transplantation, mucositis, or acute GvHD was comparable between allo-HSCT recipients with and without the detection of these two taxa. Further studies are required to clarify whether they are involved in death of recipients.

Another possible explanation for the higher mortality rate among patients positive for *Staphylococcus haemolyticus* or *Ralstonia pickettii* is that oral dysbiosis is a surrogate marker for a dysbiotic pattern of the intestinal microbiota. Intensive chemotherapy and irradiation allow bacteria to enter the systemic circulation through the disrupted mucosal barrier of the GI tract and/or oral cavity. This then provides a cytokine milieu fit for T-cell proliferation and activation and leads to the development of GvHD after donor–cell engraftment. As a previous mouse model indicated, intestinal GvHD targeted intestinal stem cells as well as their niche Paneth cells play a pivotal role in the regeneration of the damaged mucosal epithelium. The loss of Paneth cells resulted in reduced production of α-defensins, antimicrobial peptides required for maintaining intestinal microbial ecology; less diversity of the intestinal microbiome in turn had an impact on the mortality of allo-HSCT recipients [9]. In this study, we found no significant relationship between dysbiosis of the oral cavity and incidence or severity of intestinal GvHD (S4 Table). To clarify these issues, a direct comparison of the microbiome diversity, and their components, between the oral cavity and gastrointestinal tract in each case, should be performed in future studies.

These two taxa were detected from 2 of 19 patients receiving only prophylactic antibiotics, whereas they were also detected from over half of the patients receiving additional antibiotics for the treatment of neutropenic fever/infections (Table 5). Furthermore, higher detection rates were observed in the patients receiving multidrug regimens relative to those receiving additional treatment by single cephem (S3 Table). Although we could not specify the causative antibiotic/s, our results suggest that heavy treatment with multiple antibiotics during the pretransplant conditioning period strongly correlated with detection of the two taxa in the disrupted tongue microbiota.

We confirm that detection of either/both of the two taxa was significantly associated with the high risk of mortality, independent of other relevant factors such as risk status at transplantation or use of additional antibiotics. However, we could not construct a further complex model incorporating all potential confounding factors, because more than two exposures (1/10 of the number of events, 20 events in this study) should not be incorporated into the Cox proportional hazards regression model in order to avoid over-adjustment [25]. A future study with a larger sample size is needed to show that the detection of the two taxa is a completely independent predictor for mortality risk.

The tongue coating of community-dwelling adults was collected using a sampling device based on an electric toothbrush developed in our previous study [26], which enabled us to collect bacteria attached to a bonded-fiber fabric on a brush head from a 15 mm-diameter circular area on the center of the tongue dorsum. On the other hand, we had already commenced with sample collection from allo-HSCT patients before development of this device, thus their samples were collected by using a conventional cotton swab. There is little difference in the bacterial composition between the samples collected by the two devices. However, a quantity of bacteria, collected using a cotton swab, remained trapped in the swab after centrifugation. Hence, use of a cotton swab resulted in lower DNA recovery than the newly developed method. This difference in the sampling device prevents the comparison of absolute bacterial quantity between the community-dwelling adults and allo-HSCT patients in this study. A future study using the quantitative sampling device, with quantitative PCR analysis, is required to clarify whether these bacteria actually colonize and grow in allo-HSCT or only survive on the tongue dorsum of the allo-HSCT recipients.

Low diversity in the intestinal microbiota was reported to be a good predictor of high risk of mortality following allo-HSCT [9]. In contrast, no significant relationship was observed between alpha diversity (Shannon diversity index) of the tongue microbiota and incidence of transplant complications, although overall survival rate tended to be lower in subjects with lower diverse microbiota (S5 Table). This result implies that detection of specific bacterial taxa in less diverse microbiota of allo-HSCT patients would be more helpful for the prediction of mortality risk rather than microbial diversity itself.

This comprehensive analysis revealed that altered indigenous microbiota of allo-HSCT recipients was observed in the oral cavity, as previously reported in the gastrointestinal tract [5]. Lowered diversity of the oral microbiota with an increased proportion of drug-resistant bacteria uncommon in the oral cavity was observed on the day of stem cell transplantation. Among such bacterial taxa, allo-HSCT recipients with the detection of *Staphylococcus haemolyticus* and/or *Ralstonia pickettii* showed inferior transplant outcome, although the mechanisms require further investigation. Careful attention should be given to bacterial composition of the disrupted oral microbiota in allo-HSCT recipients.

## Materials and methods

### Ethics statement

All participants understood the nature of the study and provided written informed consent. The ethics committee of Kyushu University Hospital approved this study (reference no. 2019–246). All experiments were conducted in accordance with approved guidelines.

### Study population and sample collection

The study population consisted of adult patients aged 36–69 years who underwent allo-HSCT at Kyushu University Hospital from February 2016 to October 2018. Oral microbiota samples were collected from the center of tongue dorsum on the day of transplantation with a cotton swab. The collected samples were placed in 200 μl of lysis buffer containing 10 mM Tris-HCl, 1 mM EDTA, and 1% sodium dodecyl sulfate. After the swab was discarded following centrifugation, DNA extraction was performed using a bead-beating method as described previously [27]. Of the 75 patients who provided samples, 30 were excluded from the analysis due to the low quantity and/or quality of the extracted DNA causing an inability to obtain sufficient amounts of PCR amplicons for sequencing.

The microbiota composition of community-dwelling adults of a similar generation was determined in order to compare the overall microbiota composition with that of allo-HSCT recipients. This population consisted of 164 adult residents of Hisayama Town, Fukuoka, Japan who received a health examination as well as periodontal disease screenings for adults aged 40, 50, 60 and 70 years in 2016 (n = 41, 35, 53, and 35 subjects, respectively). The subjects aged 70 years were part of the study population of our previous study on the tongue microbiota of community-dwelling elderly adults aged 70 to 80 years [26]. Sample collection using a sampling device based on an electric toothbrush with a bonded-fiber fabric and DNA extraction were conducted as previously described [26]. The samples from the subjects aged 40, 50 and 60 years were collected in the same health examination and processed using the same procedure.

### Ion Torrent 16S rRNA gene sequencing analysis

The V1–V2 region of the 16S rRNA gene of 45 allo-HSCT recipients was amplified using the following primers: 8F (5'- AGA GTT TGA TYM TGG CTC AG -3') with Ion Torrent adaptor A and the sample-specific 8-base tag sequence and 338R (5'- TGC TGC CTC CCG TAG GAG T -3') with the Ion Torrent trP1 adaptor sequence and the sample-specific 8-base tag sequence. PCR amplification, purification, quantification of each amplicon and amplicon pooling were performed as previously described [28]. Emulsion PCR and enrichment of template-positive particles were performed using an Ion PGM Hi-Q View OT2 kit (Thermo Fisher Scientific, Waltham, MA), and sequencing was performed with the Ion PGM (Thermo Fisher Scientific) using an Ion PGM Hi-Q View Sequencing kit (Thermo Fisher Scientific).

The V1–V2 region of 16S rRNA gene of the 35 community-dwelling adults aged 70 years was determined in our previous study [26] with the Ion PGM (Thermo Fisher Scientific) and the same reagents used in this study, except that the previous reverse primer did not contain an 8-base tag sequence. The V1–V2 regions of 16S rRNA gene sequences of the remaining 130 residents aged 40, 50 and 60 years were determined via the same procedure.

The quality of the raw sequence reads was checked using a script written in R as previously described [26]. The quality-filtered reads were assigned to the appropriate samples based on their tag sequences, followed by trimming their adaptor, tag, and forward primer sequences. Similar sequences were assigned into operational taxonomic units (OTUs) using UPARSE [29], with a minimum pairwise identity of 97%. Following rarefaction to 2,000 reads per sample to correct for the unequal number of sequences per sample (a minimum of 2,182 reads), alpha diversity indices were calculated using the diversity function in the vegan library of R. The dissimilarity between all bacterial communities was assessed using the unweighted UniFrac metric [30], and the similarity relationship was represented in a principal coordinate analysis (PCoA) plot drawn by R. The taxonomy of representative sequences was determined using the RDP classifier with a minimum support threshold of 80% and the RDP taxonomic nomenclature (to the genus level). The taxonomy of the OTUs which were only present in allo-HSCT recipients was further searched using BLAST against 998 bacterial 16S rRNA gene sequences (HOMD 16S rRNA RefSeq version 15.1) in the expanded Human Oral Microbiome Database (eHOMD) [16] with a threshold identity of 98.5%.

### Full length 16S rRNA gene sequencing analysis using Pacbio Sequel

The 16S rRNA genes containing all variable regions were amplified using the following primers: 8F (5'- AGA GTT TGA TYM TGG CTC AG -3') with Ion Torrent adaptor A and the sample-specific 8-base tag sequence and 1492R (5'- GGY TAC CTT GTT ACG ACT T -3'). PCR amplification was carried out using KOD DNA polymerase (Toyobo, Osaka, Japan) under the following cycling conditions: 98°C for 2 min, followed by 30 cycles of 98°C for 15 s, 60°C for 20 s, and 74°C for 90 s. Equal amounts of DNA were pooled following purification using an Agencourt AMPure XP kit (Beckman Coulter, Brea, CA, USA). The pooled DNA was gel purified using a Wizard SV gel and PCR cleanup system (Promega, Madison, WI, USA). The purified amplicons were sequenced using the Sequel Sequencing kit 2.1 (Pacific Biosciences, Menlo Park, CA, USA) on a PacBio Sequel (Pacific BioSciences). The obtained long sequence reads, which repeatedly determined the template DNA, were processed using SMRT Link software version 5.1.0.26412 (Pacific Biosciences), which provided circular consensus sequence (CCS) reads with high accuracy.

The CCS reads were excluded from the analysis if they were ≤1,000 bases, if they were ≥1,700 bases, if they had an average quality score of ≤40, or if they did not include the correct forward and reverse primer sequences. The remaining CCS reads were assigned to the appropriate sample by examining tag sequences using R, followed by trimming of tag and primer sequences. The 211,164 quality-checked CCS reads were further processed by using the DADA2 pipeline [31] including the quality-filtering, denoising and chimera-filtering procedures with default settings for PacBio reads (version 1.9.1). The taxonomy of each denoised CCS sequence was determined using BLAST against 998 oral bacterial 16S rRNA gene sequences in the eHOMD (eHOMD 16S rRNA RefSeq version 15.1) [16]. Nearest neighbor taxons with ≥98.5% identity were selected as candidates for each sequence. The denoised sequences with no hit were further compared against 695,172 bacterial sequences in SILVA database (SILVA_132_SSURef_Nr99_tax_silva.fasta) and nearest neighbors with ≥98.5% identity were selected as candidates for each sequence. The taxonomy of the remaining undefined sequences was determined up to the genus level using the RDP classifier with a minimum support threshold of 80%. *Pseudomonas fluorescens* HOT-612 was uniquely identified in a slight amount of PCR amplicon obtained from negative control in our preliminary analysis, thus we excluded the sequences corresponding to this taxon from the analysis, as PCR contaminant. We also discarded the sequences corresponding to phylum *Cyanobacteria*/*Chloroplast*, because all these sequences matched with the chloroplast’s 16S rRNA genes in plants. The numbers of the denoised CCS sequences corresponding to the same taxa were combined, and the relative abundance and detection frequencies of each taxon were calculated in R. The sequence data obtained in this study have been deposited in the DDBJ Sequence Read Archive under accession no. DRA009550 and DRA009551.

### Statistical analysis

All statistical analyses were conducted with R version 3.5.1. The Wilcoxon rank-sum test was conducted to compare the alpha diversity indices between the community-dwelling adults and the allo-HSCT recipients. The relative abundance of bacterial genera were compared using a Wilcoxon rank-sum test followed by *P* value adjustment for multiple comparison. Fisher’s exact test was conducted to analyze the relationship between the presence of non-oral bacteria and incidence of transplant complications (oral mucositis, bacteremia until day 100, acute GVHD and death) during the follow-up period (by July 21, 2019; 283–1258 days). The survival curves were generated by the Kaplan–Meier method and a log-rank test was conducted to examine significant differences in survival curves between the recipients with and without specific bacterial taxa. A Cox proportional hazard regression analysis was performed to estimate adjusted hazard ratio and 95% CIs. The risk status at transplantation was classified based on the criteria described previously [32].

## Supporting information

S1 Fig Rarefaction curve for a number of OTUs in allo-HSCT patients and community-dwelling adults in the V1–V2 regions of 16S rRNA gene analysis by Ion PGM. (TIF)

S2 Fig Rarefaction curve for a number of unique sequences per samples in the full-length 16S rRNA gene analysis by PacBio Sequel. (TIF)

S3 Fig Comparison between the results of V1-V2 regions and full-length 16S rRNA gene analyses. Alpha diversity (number of identified sequences) and the relative abundances of seven predominant bacterial taxa (mean relative abundance >5%) in each analysis are shown.
(TIF)

S4 Fig Relative abundance of predominant bacterial taxa with mean relative abundances >1% and 34 non-oral taxa in the tongue microbiota of 45 allo-HSCT patients on the transplantation date in full-length 16S rRNA gene analysis using PacBio Sequel. (TIF)

S5 Fig A principal coordinate analysis plot showing similarity relationship among tongue microbiota of allo-HSCT patients who received different antibiotic use and conditioning regimens, and have different underlying diseases using an unweighted UniFrac metric, respectively. The points corresponding to different groups are depicted in different colors in each diagram. The microbiota difference between the groups were investigated statistically by permutational multivariate analysis of variance (perMANOVA) test. The ellipses cover 67% of the samples belonging to each sample type.
(TIF)

S1 Table Bacterial taxa corresponding to 12 OTUs present in the tongue microbiota of multiple allo-HSCT recipients on the transplantation date but absent in 164 community-dwelling adults (CDA) in V1-V2 regions of 16S rRNA gene sequencing data which rarified 2000 reads per sample. (PDF)

S2 Table Incidence of transplant complications in the recipients with the detection of four non-oral bacterial taxa. (PDF)

S3 Table Relationship between the detection of *Staphylococcus haemolyticus* and/or *Ralstonia pickettii* and antibiotics used during pretransplant conditioning. (PDF)

S4 Table Relationship between the detection of *Staphylococcus haemolyticus* and/or *Ralstonia pickettii* and the severity of intestinal GvHD. (PDF)

S5 Table Incidence of transplant complications in the recipients with the microbiota with different alpha diversity (Shannon diversity index). (PDF)
