# Supplementary figures and images for: Disrupted tongue microbiota and detection of nonindigenous bacteria on the day of allogeneic hematopoietic stem cell transplantation
Source: PLoS Pathog. 2020 Mar 9;16(3):e1008348. doi: 10.1371/journal.ppat.1008348 (PMC7082065; doi:10.1371/journal.ppat.1008348)

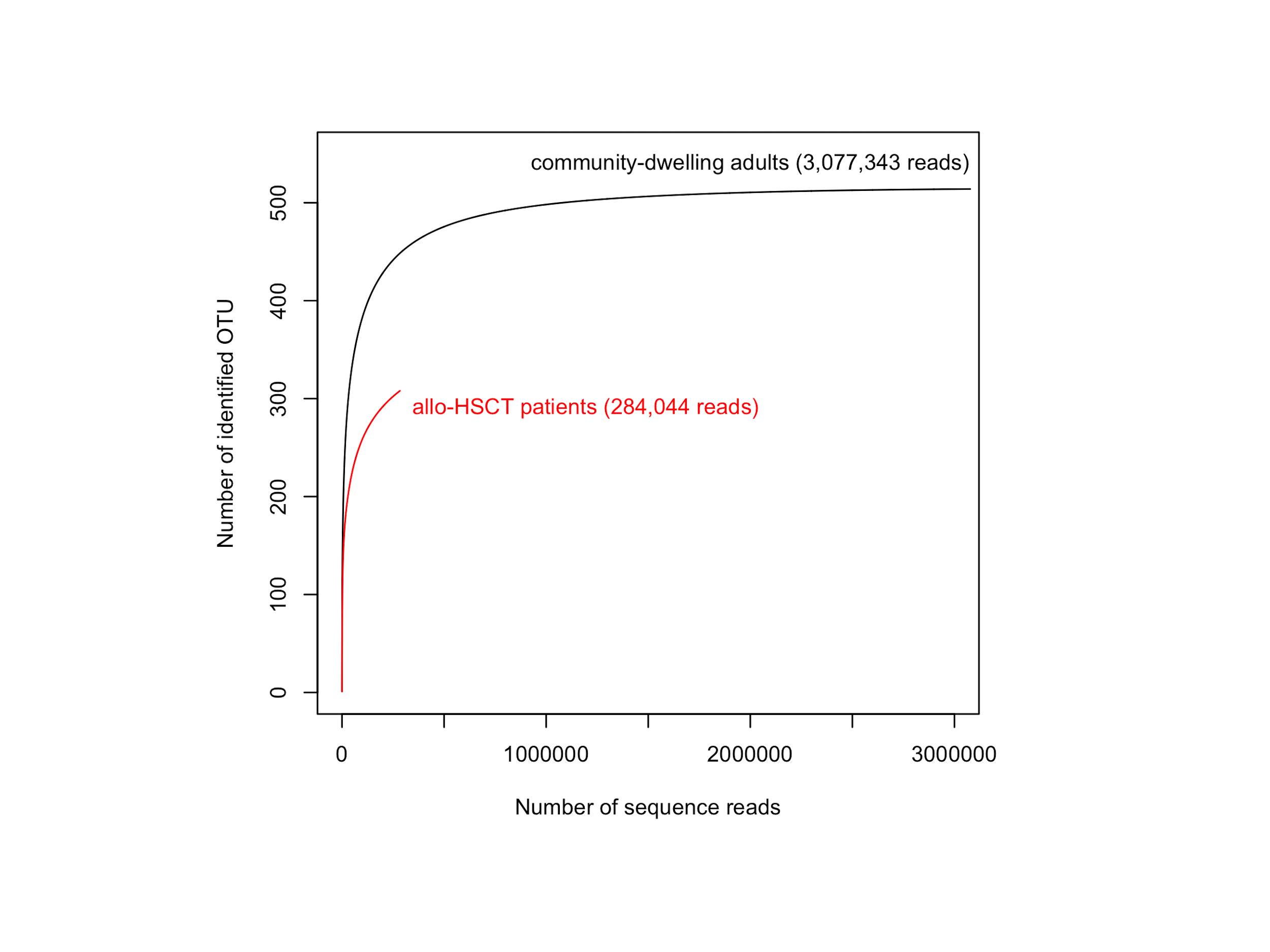

Supplement: S1 Fig — (TIF) [file ppat.1008348.s001.tif]

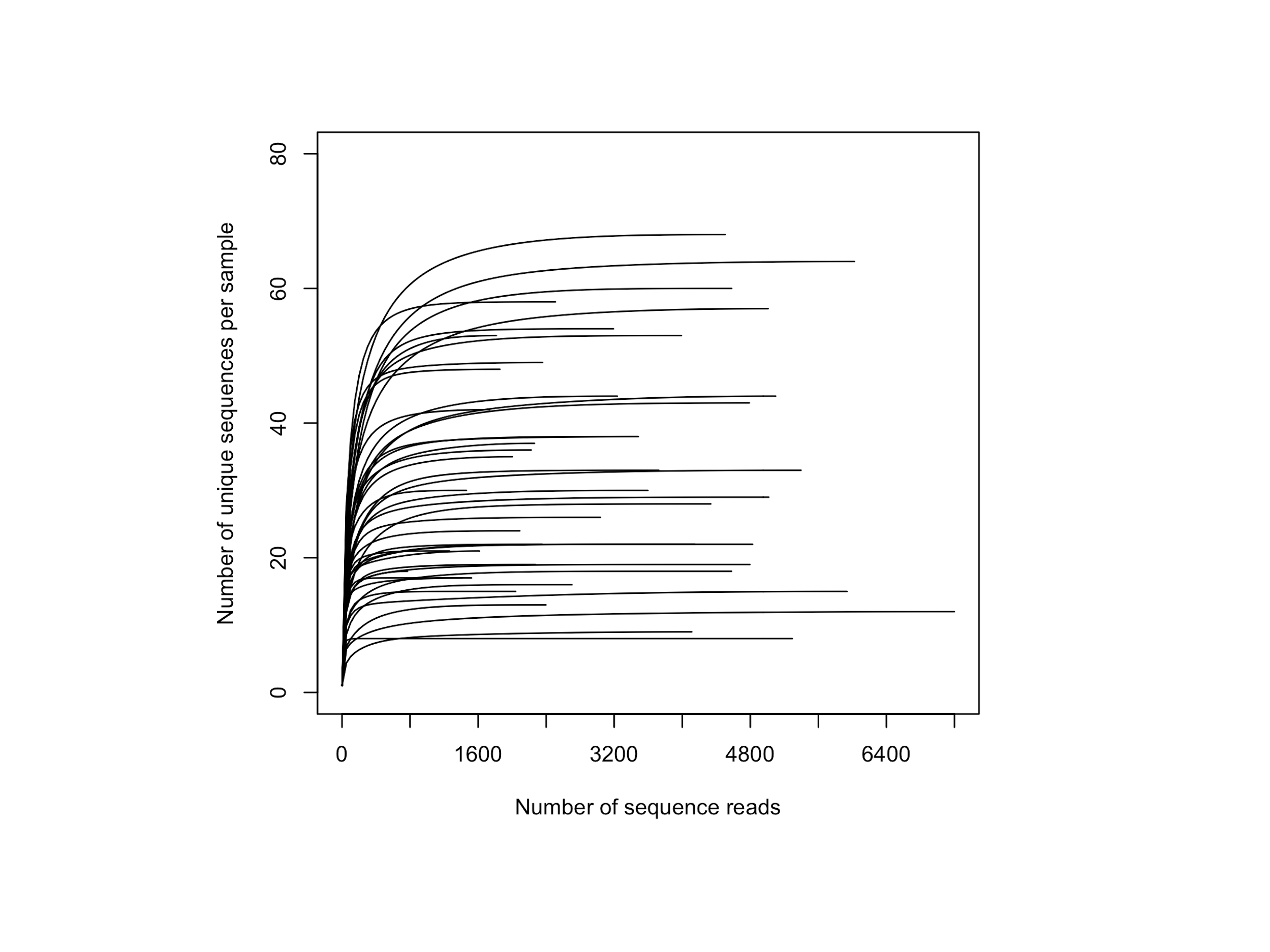

Supplement: S2 Fig — (TIF) [file ppat.1008348.s002.tif]

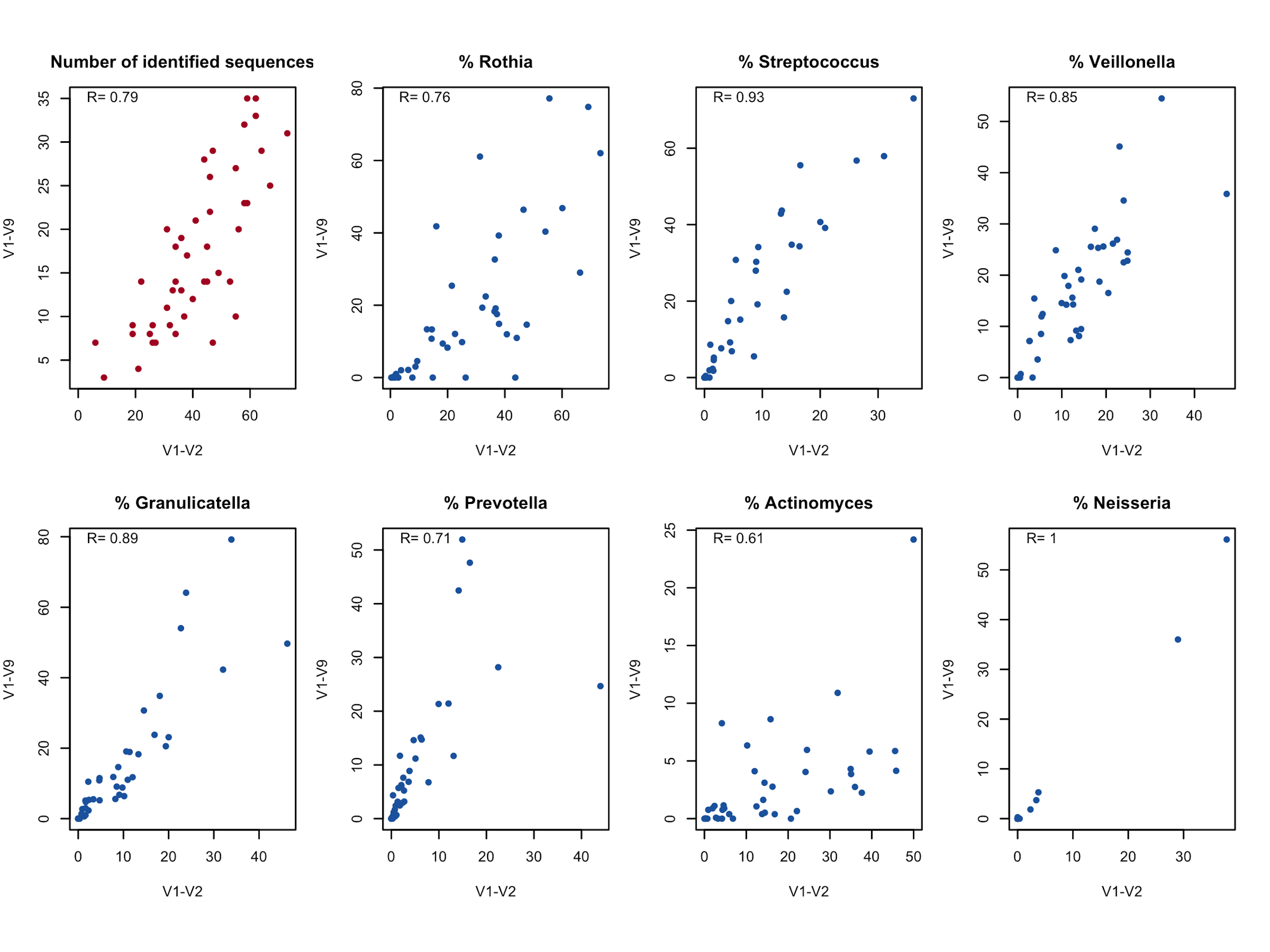

Supplement: S3 Fig — Alpha diversity (number of identified sequences) and the relative abundances of seven predominant bacterial taxa (mean relative abundance >5%) in each analysis are shown. (TIF) [file ppat.1008348.s003.tif]

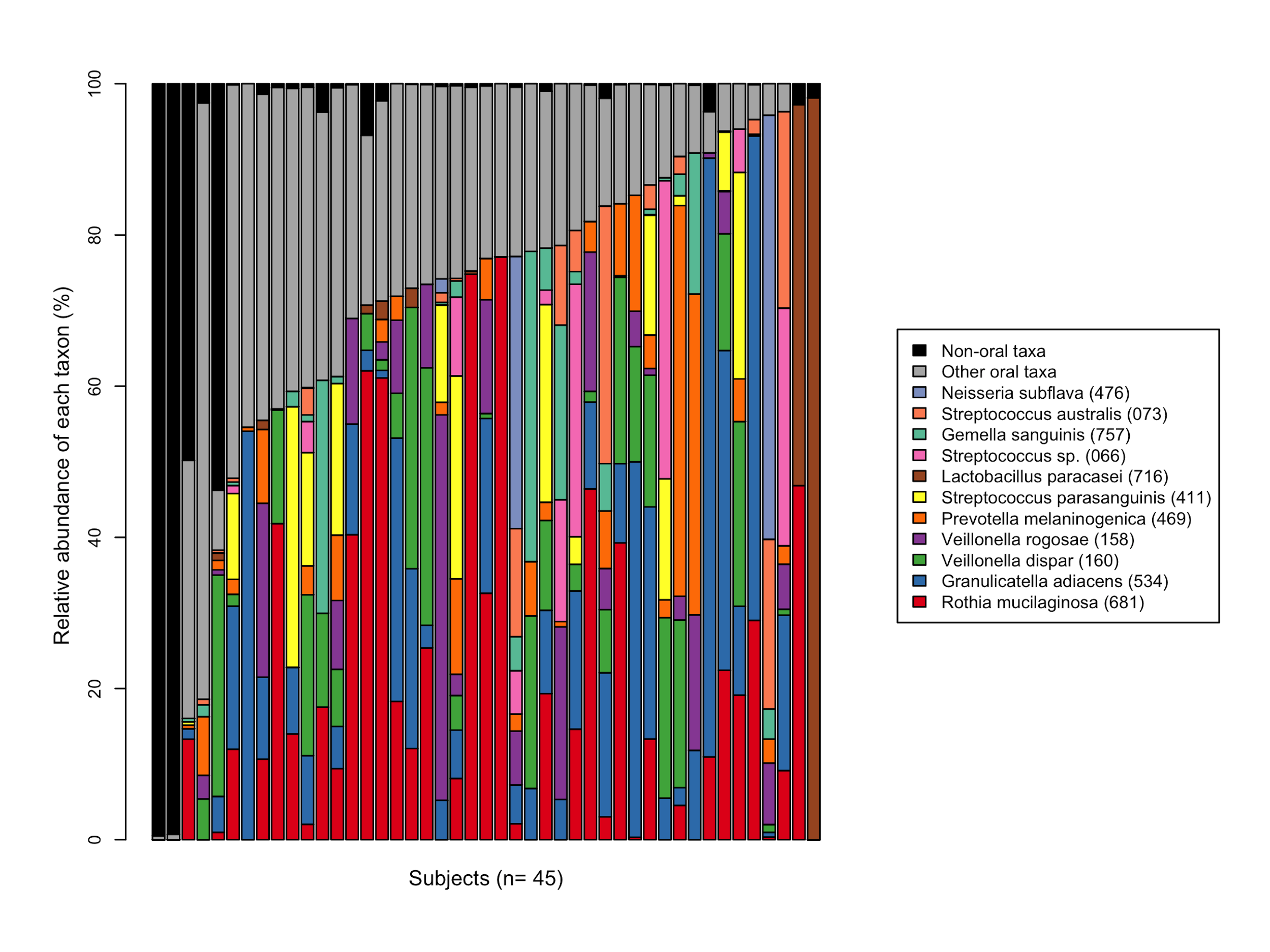

Supplement: S4 Fig — (TIF) [file ppat.1008348.s004.tif]

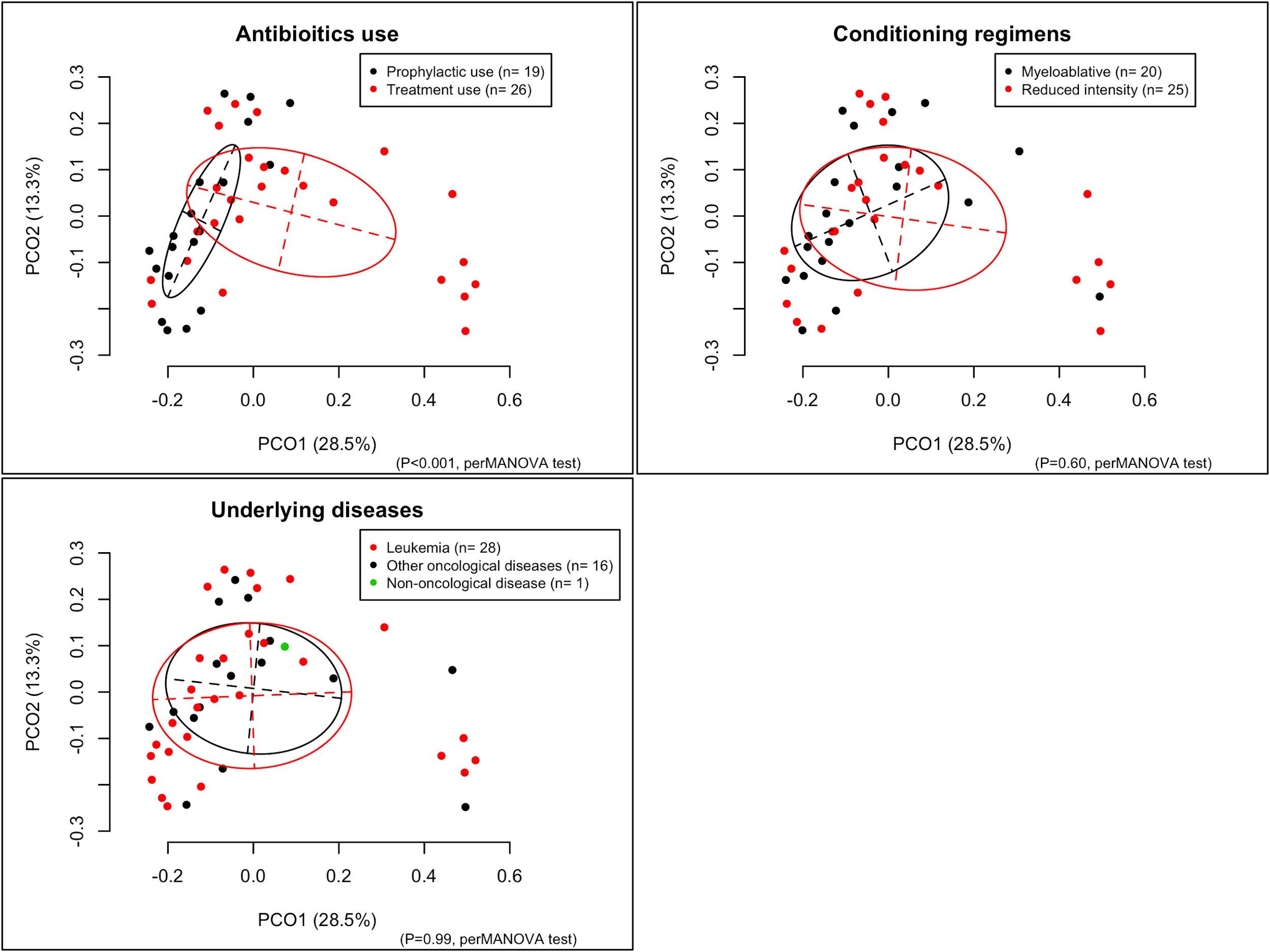

Supplement: S5 Fig — The points corresponding to different groups are depicted in different colors in each diagram. The microbiota difference between the groups were investigated statistically by permutational multivariate analysis of variance (perMANOVA) test. The ellipses cover 67% of the samples belonging to each sample type. (TIF) [file ppat.1008348.s005.tif]
