# Supplementary material for: Disrupted tongue microbiota and detection of nonindigenous bacteria on the day of allogeneic hematopoietic stem cell transplantation
Source: PLoS Pathog. 2020 Mar 9;16(3):e1008348. doi: 10.1371/journal.ppat.1008348 (PMC7082065; doi:10.1371/journal.ppat.1008348)
Supplement: S1 Table — (PDF) [file ppat.1008348.s006.pdf]

S1 Table. Bacterial taxa corresponding to 12 OTUs present in the tongue microbiota of multiple allo-HSCT recipients on the transplantation date but absent in 164 community-dwelling adults (CDA) in V1-V2 regions of 16S rRNA gene sequencing data which rarified 2000 reads per sample.

OTU No.	No. of recipients with each OTU	Relative abundances (%) Mean	Max	Bacterial taxa corresponding to each OTU (taxon ID)[a]	Body sites designated in eHOMD[b]	No. of reads in total reads from CDA[c]
#39	15	2.60 ± 14.41	95.6	*Lactobacillus paracasei* (716)	Oral	0
#20	10	3.86 ± 14.56	64.8	*Staphylococcus haemolyticus* (120)/*hominis* (127)	nonOral/nonOral	1
#97	10	0.06 ± 0.23	1.5	*Ralstonia pickettii* (716)/sp. (406)	nonOral/nonOral	0
#57	9	0.25 ± 1.06	5.1	Genus *Xanthomonas*	No hit	0
#222	5	0.01 ± 0.03	0.1	Genus *Clostridium sensu stricto*	No hit	0
#103	3	0.18 ± 0.88	5.3	*Enterococcus durans* (880)	nonOral	0
#162	3	0.03 ± 0.17	1.1	*Lactobacillus rhamnosus* (749)/*casei* (568)	Oral/nonOral	0
#56	3	0.52 ± 3.50	23.5	*Corynebacterium tuberculostearicum* (077)	nonOral	0
#95	2	0.02 ± 0.09	0.5	Genus *Acinetobacter*	No hit	1
#201	2	0.02 ± 0.11	0.7	*Enterococcus faecalis* (604)	nonOral	0
#495	2	0.00 ± 0.02	0.1	Genus *Capnocytophaga*	No hit	0
#50	2	0.41 ± 2.74	18.3	Genus *Pseudomonas*	No hit	2

Values with errors are mean ±SD. [a]Taxon ID in expanded Human Oral Microbiome database (eHOMD) is given in parentheses following bacterial name. [b] 'nonOral' indicates that 'Oral' is not included in the 'Body Site' status of the species in eHOMD. 'No hit' indicates that no candidate species corresponding to the OTU were found in eHOMD database. [c]The number of reads corresponding to each OTU in all 3,077,343 reads obtained from the community-dwelling adults.
