# Supplementary material for: Disrupted tongue microbiota and detection of nonindigenous bacteria on the day of allogeneic hematopoietic stem cell transplantation
Source: PLoS Pathog. 2020 Mar 9;16(3):e1008348. doi: 10.1371/journal.ppat.1008348 (PMC7082065; doi:10.1371/journal.ppat.1008348)
Supplement: S2 Table — (PDF) [file ppat.1008348.s007.pdf]

S2 Table. Incidence of transplant complications in the recipients with the detection of four non-oral bacterial taxa.

Bacterial taxa[a]	No. of subjects	No. (%) of recipients with transplant complications[b] Oral mucositis[c]	Bacteremia until day+100	Acute GvHD[d]	One-year overall survival rate (%) (95% CI)
Ralstonia pickettii (854)					
Detected	13	7 (53.8)	5 (38.4)	5 (38.5)	32.3 (13.4–77.5)
Not detected	32	21 (65.6)	7 (21.8)	10 (31.2)	74.7 (61.0–91.5)
Staphylococcus haemolyticus (120)					
Detected	8	4 (50.0)	4 (50.0)	3 (37.5)	37.5 (15.3–91.7)
Not detected	37	24 (64.9)	8 (21.6)	12 (32.4)	69.3 (55.7–86.3)
Uncultured organism (HQ813300)					
Detected	8	7 (87.5)	1 (12.5)	1 (12.5)	85.7 (63.3–100)
Not detected	37	21 (56.8)	11 (29.7)	14 (37.8)	59.0 (44.9–77.4)
Streptococcus thermophilus (152)					
Detected	6	3 (50.0)	3 (50.0)	1 (16.6)	41.7 (14.6–100)
Not detected	39	25 (64.1)	9 (23.0)	14 (35.9)	66.5 (53.1–83.1)

Only four non-oral taxa identified from more than five recipients were shown. [a]Taxon ID in expanded Human Oral Microbiome database (triple digits) or GenBank number is given in parentheses following bacterial name. [b]No significant relationship was observed between the presence of each taxon and each complication in Fisher's exact test. [c]Grade 0 is regarded as absence of oral mucositis. [d]Grade II-IV is regarded as presence of acute GvHD.

Abbreviation: GvHD, graft-versus host disease
