# Supplementary material for: Disrupted tongue microbiota and detection of nonindigenous bacteria on the day of allogeneic hematopoietic stem cell transplantation
Source: PLoS Pathog. 2020 Mar 9;16(3):e1008348. doi: 10.1371/journal.ppat.1008348 (PMC7082065; doi:10.1371/journal.ppat.1008348)
Supplement: S3 Table — (PDF) [file ppat.1008348.s008.pdf]

S3 Table. Relationship between the detection of *Staphylococcus haemolyticus* and/or *Ralstonia pickettii* and antibiotics used during pretransplant conditioning.

	Detection of bacterial taxa	
	Staphylococcus haemolyticus (n= 8)	*Ralstonia pickettii* (n= 13)
Prophylactic use (n= 19)	0 (0)	2 (15.3)
Additional use for treatment (n= 26)	8 (30.7)	11 (84.6)
CFPM (n= 14)	2 (14.2)	4 (28.4)
CZOP (n= 1)	0 (0)	1 (100.0)
Other antibiotics (n= 11)	6 (54.5)	6 (54.5)
DRPM (n=1)	1 (100)	0 (0)
CFPM+TAZ/PIPC (n= 2)	1 (50)	1 (50)
CZOP+TAZ/PIPC (n= 1)	1 (100)	1 (100)
DRPM+TAZ/PIPC (n= 1)	0 (0)	0 (0)
CFPM+TAZ/PIPC+DRPM (n= 1)	0 (0)	1 (100)
CFPM+TAZ/PIPC+TEIC (n= 1)	1 (100)	1 (100)
MEPM+TEIC (n= 1)	0 (0)	1 (100)
TAZ/PIPC+MEPM+TEIC (n= 2)	2 (100)	1 (50)
CFPM+DRPM+VCM (n= 1)	0 (0)	0 (0)

CFPM, cefepime; CZOP, cefozopran; DRPM, doripenem; MEPM; meropenem; TAZ/PIPC; tazobactam/piperacillin; TEIC; teicoplanin; VCM; vancomycin.
