# Supplementary material for: Disrupted tongue microbiota and detection of nonindigenous bacteria on the day of allogeneic hematopoietic stem cell transplantation
Source: PLoS Pathog. 2020 Mar 9;16(3):e1008348. doi: 10.1371/journal.ppat.1008348 (PMC7082065; doi:10.1371/journal.ppat.1008348)
Supplement: S4 Table — (PDF) [file ppat.1008348.s009.pdf]

S4 Table. Relationship between the detection of *Staphylococcus haemolyticus* and/or *Ralstonia pickettii* and the severity of intestinal GvHD.

	Either/both of the two taxa		
	Detected (n= 17)	Not detected (n= 28)	*P*-value[a]
Severity of intestinal GvHD			0.742
Grade 0	14 (82.3)	18 (64.2)	
Grade 1	2 (11.7)	7 (25.0)	
Grade 2	1 (5.8)	1 (3.5)	
Grade 3	0 (0)	2 (7.1)	

[a]Fisher's exact test.

Abbreviation: GvHD, graft-versus host disease
