# Supplementary material for: Disrupted tongue microbiota and detection of nonindigenous bacteria on the day of allogeneic hematopoietic stem cell transplantation
Source: PLoS Pathog. 2020 Mar 9;16(3):e1008348. doi: 10.1371/journal.ppat.1008348 (PMC7082065; doi:10.1371/journal.ppat.1008348)
Supplement: S5 Table — (PDF) [file ppat.1008348.s010.pdf]

S5 Table. Incidence of transplant complications in the recipients with the microbiota with different alpha diversity (Shannon diversity index).

| | Shannon diversity index | | |
| --- | --- | --- | --- |
| | Low tertile (n= 15) | Mid tertile (n= 15) | High tertile (n= 15) |
| Oral mucositis[a,c] | 7 (46.6) | 10 (66.6) | 11 (73.3) |
| Bacteremia until day+100 [c] | 4 (26.6) | 3 (20.0) | 5 (33.3) |
| Acute GvHD[b,c] | 5 (33.3) | 4 (26.6) | 6 (40.0) |
| One-year overall survival rate (%)[d] | 53.3 | 64.0 | 72.7 |
| (95% CI) | (33.2–85.6) | (42.9–95.5) | (53.1–99.6) |

[a]Grade 0 is regarded as absence of oral mucositis. [b]Grade II-IV is regarded as presence of acute GvHD. [c]No significant relationship was observed in Fisher's exact test. [d]No significant relationship was observed in a log-rank test.

Abbreviation: GvHD, graft-versus host disease
